# Endurance exercise training decreased serum levels of surfactant protein D and improved aerobic fitness of obese women with type-2 diabetes

**DOI:** 10.1186/s13098-017-0273-6

**Published:** 2017-09-25

**Authors:** Sajjad Rezaei, Mahdieh Molanouri Shamsi, Mehdi Mahdavi, Azadeh Jamali, Jonato Prestes, Ramires Alsamir Tibana, James Wilfred Navalta, Fabrício Azevedo Voltarelli

**Affiliations:** 1 Physical Education and Sport Sciences Department, Faculty of Humanities, Tarbiat Modares University, Jala Ale Ahmad Exp. Tehran, P.O.Box: 14117-13116, Tehran, Iran; 2 Immunology Department, Pasteur Institute of Iran, Tehran, Iran; 3 Department of Physical Education, Taft Branch, Islamic Azad University, Taft, Iran; 4 Graduate Program in Health Sciences and Technologies, University of Brasilia, Brasilia, Brazil; 5 Department of Kinesiology and Nutrition Sciences, University of Nevada, Las Vegas, NV USA; 6 Graduation Program of Physical Education, Faculty of Physical Education, Federal University of Mato Grosso, Cuiabá, Brazil

**Keywords:** Surfactant protein D, Exercise training, Type-2 diabetes *mellitus*, Obesity

## Abstract

**Background:**

Surfactant protein D (SP-D) is a member of the collectin family and is an important component of the pulmonary innate host defense. To find the relationship between exercise training and SP-D in diabetes, we examined the possible effects of a 10-week endurance exercise-training program on serum levels of SP-D, leptin, lipid profile and insulin resistance in obese women with type-2 diabetes *mellitus* (T2DM).

**Methods:**

Twenty-two obese women with T2DM were randomly assigned to either exercise training (ET) or control (C) group. A subject dropped from ET group due to personal reasons and 1 subject dropped from C group due to commitment to experiments conditions. A total of twenty obese women with T2DM were randomly assigned into endurance exercise training (ET = 10) and control (C = 10) groups. The training group underwent a progressive endurance-training program for 10 weeks (running on a treadmill for 30–55 min/day at 50–75% heart rate reserve) and the control group did not participate in any exercise program. Venous blood samples were collected from both groups before and 72 h after the last session of exercise training for analysis of serum SP-D, leptin, lipid profile, glucose and insulin. Data were analyzed using 2 (group: control, endurance training) × 2 (time: pre, post) ANOVA with repeated measures on the second factor. Absolute changes from rest (∆ baseline) values were calculated according to the following formula: ((measure-baseline)·baseline-1)·100. Percent change between groups was analyzed using independent t-tests (p < 0.05). All analyses were completed using SPSS 19.

**Results:**

The serum SP-D levels were decreased after exercise training in ET (∆ = − 78.78 ± 17.14%, p = 0.001) when compare to C (∆ = 9.41 ± 4.75%). Obese diabetic women in the ET group showed significantly lower serum leptin levels (8053.27 ± 878.7 pg/ml, ∆ = − 26.97 ± 16.41%) when compared with women in the control group (9885.5 ± 696 pg/ml, ∆ = 7.02 ± 3.46%, p = 0.003). Fasting glucose was favorably and significantly affected by the intervention (ET ∆ = − 17.01 ± 12.74%, Control ∆ = 15.47 ± 7.32%, p = 0.011). VO_2max_ as an index of aerobic fitness was significantly increased after 10-weeks of endurance exercise training (ET ∆ = 19.29 ± 6.18%).

**Conclusions:**

Endurance exercise training with improvement in aerobic fitness induced a significant reduction of serum SP-D levels in obese women with T2DM.

## Background

Obesity is one of the most common health problems the world today is facing and has reached epidemic proportions. Obesity is associated with a risk of developing other metabolic diseases including type-2 diabetes *mellitus* (T2DM) [1, 2]. T2DM is associated with recruitment and/or activation of innate immune cells and studies support the hypothesis that this disease is caused by activated innate immunity [3, 4].

Surfactant protein D (SP-D) is a lung-specific protein that has major anti-microbial and anti-inflammatory effects [5–7]. It is detectable in human serum by specific assay [8], but the function of SP-D in serum still remains controversial. Increased SP-D serum levels were associated with cardiovascular disease-related mortality [9]. In contrast, Systemic SP-D has been shown previously to decrease in T2DM, be positively associated with insulin sensitivity [10], and negativity associated with obesity [11, 12]. As pulmonary function decline has recently been proposed as a novel risk factor for glucose intolerance, insulin resistance and T2DM [10], there is a possible that decreasing serum SP-D protein in T2DM is connected with obesity, insulin resistance and inflammation [10]. However, the specific mechanism of the relation between SP-D and metabolic disturbances, and T2DM in particular, is not clear.

Physical exercise represents an effective strategy to prevent and/or treat T2DM [13, 14]. In obese T2DM patients the focus is generally on the implementation of endurance-type exercise training. Endurance exercise training has been shown to improve metabolic outcomes in obesity and T2DM in human studies. The beneficial effects of endurance exercise training include weight loss [15] as well as improvement of insulin sensitivity and glucose metabolism [16].

Only one human study has verified the effect of exercise training on SP-D levels. Christensen et al. reported that SP-D decreased in serum following an acute session of physical exercise in individuals with rheumatoid arthritis disease [17]. Also, Fernandez-Real et al. showed that body weight reduction through diet resulted in a significant decrease in plasma SP-D concentrations [10].

On account of the high prevalence of diabetes *mellitus*, further investigation to identify suitable strategies in order to prevent and/or to slow the progression of the disease is of great importance. Given the known positive effects of aerobic training on body mass, insulin sensitivity and glucose metabolism in obese people with T2DM, and taking into consideration the association between SP-D and these parameters, we can suggest that exercise was capable to induce positive alterations in SP-D serum levels of the subjects. To the best of our knowledge, the effect of aerobic training on plasma SP-D levels in subjects with T2DM has not been investigated. The purpose of the current study was to investigate the effect of a 10-week aerobic training program on plasma SP-D levels, insulin resistance index and anthropometric and functional characteristics of obese women with T2DM.

## Methods

### Study design, subjects and intervention

We used a convenience approach for selecting subjects with T2DM [18] from the Motahari Polyclinic of Shiraz University of Medical Sciences in Shiraz, Iran. Initially, medical records of about 800 women with T2DM who were registered in Motahari Polyclininc (until July 2014) were screened. Patients were eligible for the study if they were pre-menopausal women (aged 30–45 years) with a body mass index of ≥ 30, in a good health, without recent illnesses and cardiovascular disease, none insulin use, were nonsmokers, having primary school education or higher, having no background of committing suicide, mental disorders, or drug abuse, live sedentary (≤ 1 exercise session per week), none had performed any formal exercise training in the preceding 6 months, none had weight loss diet program and living in Shiraz. From an initial eligible subject pool of 50 individuals, 22 agreed to take part in the study. Subjects completed a medical questionnaire and gave written informed consent. Immediately after finishing screen, twenty-two subjects were randomly assigned to either exercise training (ET) or control (C) group. A subject dropped from ET group due to personal reasons and 1 subject dropped from C group due to commitment to experiments conditions. There were 10 subjects in ET group and 10 subjects in C group (Table 1). The experimental procedure was going through a formal process at Tarbiat Modares University and Motahari Polyclininc. It was approved by Research Ethics Committee of Tarbiat Modares University and received a permission from Motahari Polyclininc of Shiraz University of Medical Sciences in Shiraz (Local permission number: 92/H/2477-2013/06/22).

**Table 1:** Body composition and biological parameters of study subjects before and after the exercise program

	C			ET		
	Before	After	p-value	Before	After	p-value
Age (years)	43.5 ± 2.8			42 ± 3.6		
Weight (kg)	68.6 ± 2.31	71.34 ± 2.68	0.119	72.96 ± 2.39	*69.32* *±* *1.91*	*0.097*
Height (m)	1.546 ± 3.1	1.546 ± 3.1	–	1.57.2 ± 6.4	1.57.2 ± 6.4	–
BMI (Kg/m^2^)	34.94 ± 1.09	36.32 ± 1.26	0.112	35.71 ± 0.86	*33.99* *±* *0.92*	*0.092*
Waist circumstance (cm)	91.7 ± 3.06	93.98 ± 3.12	0.398	91.75 ± 1.9	*87.85* *±* *1.87*	*0.006*
WHR	0.84 ± 0.2	0.838 ± 0.2	0.945	0.869 ± 0.25	*00.825* *±* *0.22*	*0.370*
Fasting glucose (mg/dl)	138.8 ± 6.69	173.3 ± 15.8	0.075	130.7 ± 8.67	*120.7* *±* *11.23* ^a^	*0.575*
Insulin (IU/l)	13.27 ± 1.08	13.3 ± 1.11	0.948	12.9 ± 0.85	*12.47* *±* *0.98*	*0.321*
HOMA-IR	4.52 ± 0.46	5.54 ± 0.48	0.075	4.22 ± 0.51	*3.72* *±* *0.42* ^a^	*0.383*
TG (mg/dl)	135.4 ± 10.17	145.5 ± 10.02	0.228	135.7 ± 10.2	*122.44* *±* *10.89*	*0.080*
TC (mg/dl)	160.10 ± 6.7	166.6 ± 10.6	0.668	157 ± 8.14	*150.07* *±* *7.81*	*0.638*
HDL-C (mg/dL)	48.6 ± 0.93	49.4 ± 1.19	0.502	47.2 ± 0.78	*49.4* *±* *1.76*	*0.502*
LDL-C (mg/dl)	84.7 ± 5.8	84.0 ± 2.8	0.895	83.1 ± 4.1	*76* *±* *4.2*	*0.029*
VO2max (ml/kg/min)	18.86 ± 3.6	19.71 ± 4.1	0.579	20.47 ± 4.3	*25.5* *±* *1.2* ^b^	*0.011*

*BMI* body mass index, *HOMA-IR* Homeostatic Model Assessment-Insulin Resistance, *WHR* Waist-to-hip ratio, *TG* Triglycerides, *TC* Total Cholesterol, *LDL-C* Low Density Lipoprotein, *HDL-C* High Density Lipoprotein

Data are presented as mean ± S.E, p ≤ 0.05

^a^Indicate significant differences within C and ET groups

^b^Represent significant differences between before and after exercise training in each group, N = 10 obese diabetic women per group

Nearly a month after allocating subjects (August 2014), exercise training was started. The exercise training protocol consisted of 10-weeks of aerobic walking/running at 50–75% heart rate reserve (HRR) on a treadmill. Subjects were asked to engage in three supervised sessions per week in a fitness facility, while the control group received no intervention. The intensity and duration of the exercise session was monitored using of heart rate monitors (Polar Electro Oy, Finland). Each exercise session began with a warm-up protocol comprising 10 min of brisk walking, stretching and jogging at 40% HRR, and finished with cooling down by stretching for 10 min. The exercise prescription was ramped as follows: initial duration and intensity began with 30 min at 50% HRR, and increased by 5 min every 2 weeks and by 5% HRR every week. All of subjects in ET group finished the exercise training program at the same time. Subjects were instructed not to change their dietary habits for the duration of the intervention.

### Outcome measures

Weight and height were measured with a calibrated digital scale (Seca 644 handrail scale, Seca Corp, Hanover, Maryland) and an electronic stadiometer (Seca 245 measuring rod, Seca Corp). Body mass index (BMI, kg/m^2^) was calculated as weight (kg) divided by square height (m). Waist and hip circumference was measured for the determination of waist to hip ratio (WHR) using a non-stretching tape while the subject was standing erect. Cardiovascular fitness was assessed using a graded aerobic treadmill testing was undertaken by an experienced exercise physiologist and athletic therapist under the supervision of the cardiologist. Using a modified version of the standardized treadmill-based Balke protocol [18], patients performed an incremental series of exercises to the point of symptom onset or exacerbation or until maximal exhaustion was achieved [defined as a rating of perceived exertion (RPE) of 18–20 on the Borg scale] [19]. Specifically, patients began walking on the treadmill at a speed of 3.2 mph and 0% grade. The grade was increased by 1% per minute for the first 15 min, after which time the speed was increased 0.2 mph per minute. Patients were asked to rate their symptoms and RPE every minute. Each patient underwent continuous HR monitoring throughout the test. Blood pressure was assessed immediately following test termination and again following a 5-min cooldown period. The test score is the time taken on the test, in minutes. Ideally this should be between 9 and 15 min. The test time was converted to an estimated VO2max score using the following formula where the value “T” is the total time completed (expressed in minutes and fractions of a minute e.g. 9 min 15 s = 9.25 min): VO2 max = 1.38 (T) + 5.22.

As serum SP-D exhibits diurnal pattern and reaches to the highest levels in the morning hours [10, 17], we tried to standardize blood collecting condition by drawing blood sample between 7 a.m. and 8 a.m. from the antecubital vein with subjects in a fasted state in both ET and C groups 24 h prior to training program initiation and 72 h after the last exercise session in environmentally controlled conditions. Blood samples were centrifuged for 15 min at 3000 rpm, and serum was separated and stored at − 80 °C for later analysis. Eight days after preparation of blood sample, concentrations of total cholesterol (TC), triglyceride (TG) and high-density lipoproteins–cholesterol (HDL-c) were determined through enzymatic colorimetric assays with cholesterol esterase, cholesterol oxidase and glycerolphosphate oxidase. Serum glucose concentration was determined via a commercially available kit (Pars Azmon kit, Iran) using an enzymatic colorimetric test with glucose oxidase. LDL-c was subsequently calculated according to the formula developed by Friedewald et al. [8].

Serum SP-D, leptin and insulin were measured using specific human enzyme-linked immunosorbent assay (ELISA) kits. The SP-D assay (SP-D human ELISA kit, Aviscera Bioscience INC., California, USA) had an intra-assay coefficient of variation of 4–6% and its sensitivity amount was 30 pg/ml. The leptin ELISA kit assay (Quantikine Leptin; R&D Systems, USA) had an intra-assay coefficient of variation of 10% and its minimum measurable amount was 31.2 pg/l. Insulin concentrations were measured in serum using a commercially available kit (Diaplus Q-1, China). Insulin resistance was obtained using the homeostatic model assessment (HOMA = fasting insulin × fasting glucose/22.5) [10].

### Statistical analysis

Data are presented as mean ± SE. Normality of distribution was confirmed using Kolmogorov–Smirnov test. Data were analyzed using 2 (group: control, endurance training) × 2 (time: pre, post) ANOVA with repeated measures on the second factor. Absolute changes from rest (∆ baseline) values were calculated according to the following formula: ((measure-baseline)·baseline-1)·100. Percent change between groups was analyzed using independent t-tests. A *p* value of 0.05 or less was considered statistically significant. All analyses were completed using SPSS 19.

## Results

**Fig. 1:**
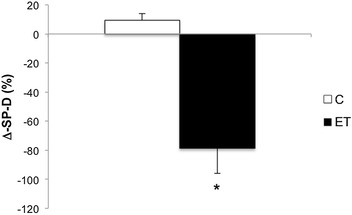
Effects of endurance training on serum levels of SP-D in control and intervention groups. Results are presented as mean ± standard deviation (SD) of the percentage change from baseline (∆). *Indicates significant difference between groups (p = 0.001)

A significant Group × Time interaction was present for serum SP-D, F (1, 18) = 25.02, p = 0.001. Groups were similar at baseline [86.5 ± 24 (ng/ml) in C group versus 85.6 ± 15 (ng/ml) in ET group, p > 0.05], however SP-D increased in the control group (∆ = 9.41 ± 4.75%), and decreased in the endurance exercise group following the intervention (∆ = − 78.78 ± 17.14%, p = 0.001, see Fig. 1).

**Fig. 2:**
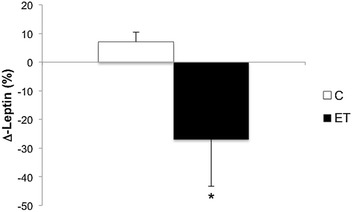
Effects of endurance training on serum levels of leptin in control and intervention groups. Results are presented as mean ± standard deviation of the percentage change from baseline (∆). *Indicates significant difference between groups (p = 0.003)

With respect to serum leptin, no interaction (F = 2.28, p = 0.149) or main effect for time (F = 0.13, p = 0.719) was noted. However, when the absolute change scores were evaluated, obese diabetic women in the ET group showed significantly lower serum leptin levels (8053.27 ± 878.7 pg/ml, ∆ = − 26.97 ± 16.41%) when compared with women in the control group who did not receive endurance training (9885.5 ± 696 pg/ml, ∆ = 7.02 ± 3.46%, p = 0.003) (see Fig. 2).

The changes in body composition and biological parameters before and after the study period in both groups are shown in Table 1. Before the intervention, there were no significant differences in age, weight, BMI, waist circumstance and WHR between the C and ET groups (p > 0.05). A significant Group × Time interaction as present for weight, F (1, 18) = 6.37, p = 0.021, with the control group increasing weight during the course of the intervention (∆ = 3.50 ± 2.18%), and the endurance trained group decreasing weight as a result of the intervention (∆ = − 5.44 ± 2.77%). However, post hoc analyses no significant differences (p = 0.21). A similar pattern was observed with respect to BMI (Group × Time interaction, F (1, 18) = 6.65, p = 0.019) however post hoc analyses revealed no significant differences (p = 0.15) (Control ∆ = 3.50 ± 2.18%, ET ∆ = − 5.44 ± 2.77%). A significant Group × Time interaction was observed for waist girth, (F (1, 18) = 4.92, p = 0.04). Waist circumstance decreased 3.9 ± 1.07 cm (∆ = − 4.53 ± 1.28%) after the intervention period in the ET group (p ≤ 0.05), but increased in the control group (∆ = 2.02 ± 2.93%). No interactions or main effects were noted for hip girth (F (1, 18) = 1.95, p = 0.179) or waist-to-hip ratio (F (1, 18) = 0.23, p = 0.63).

With regard to the metabolic profile, there were no interactions for fasting glucose (F (1, 18) = 3.41, p = 0.08), insulin (F (1, 18) = 0.72, p = 0.409), or HOMA-IR (F (1, 18) = 4.17, p = 0.056). However, when the absolute change scores were evaluated, fasting glucose was favorably and significantly affected by the intervention (ET ∆ = − 17.01 ± 12.74%, Control ∆ = 15.47 ± 7.32%, p = 0.011). Additionally, the change in HOMA-IR following the endurance exercise protocol was significantly affected (ET ∆ = − 21.45 ± 12.93%, Control ∆ = 15.75 ± 7.21%, p = 0.032).

A significant Group × Time interaction as present for serum triglycerides, F (1, 18) = 5.15, p = 0.036, with the control group increasing during the course of the intervention (∆ = 4.50 ± 5.04%), and the endurance trained group decreasing triglycerides as a result of the intervention (∆ = − 14.48 ± 7.71%). However, post hoc analyses no significant differences (p = 0.19). No interactions or main effects were noted for total cholesterol (F (1, 18) = 0.43, p = 0.521), HDL-c (F (1, 18) = 0.52, p = 0.479), or LDL-c (F (1, 18) = 1.203, p = 0.287).

No interaction was observed for VO2max (F = 3.76, p = 0.068). Although the absolute change from baseline was large for the exercise group (∆ = 19.29 ± 6.18%), statistical comparison to the control group (∆ = 1.79 ± 6.64%) yielded no significant differences (p = 0.687) (see Table 1).

## Discussion

This study aimed to test whether 10 weeks of endurance exercise training would have an effect on a specific lung innate immune protein (SP-D) in obese women with T2DM. As exercise has been shown to have a beneficial effect on both T2DM and SP-D, we hypothesized that a 10-week aerobic training program would decrease SP-D in our study population. Our hypothesis was confirmed, and we found that surfactant protein D decreased by approximately 40% as obese women with T2DM adhered to the endurance training protocol.

Surfactant protein D is a member of the surfactant proteins family including SP-A, SP-B, SP-C and SP-D. SP-D plays an important role in controlling pulmonary infections, allergies and inflammation. These proteins neutralize alveolar pathogens through activation of neutrophils and macrophages and consequent induction of phagocytosis and superoxide free radical production [5]. SP-D also contributes to the innate immune response through lysis of inspiratory pathogens and attenuates pulmonary inflammation by inhibiting surface macrophages [20].

It is well established that an abnormality in pulmonary function could result in low-grade chronic inflammation, leading to increased insulin resistance and precipitating T2DM [4]. On the other hand, obesity and metabolic abnormalities are considered as risk factors for respiratory symptoms of pulmonary disease [4, 21]. There is accumulating evidence that SP-D is at the intersection of inflammation, obesity and insulin resistance [10, 12, 22–24].

The current experiment revealed that endurance exercise training significantly decreased serum SP-D compared to baseline values. To the best of our knowledge, we are the first to report the beneficial effect of endurance training on systemic SP-D in obese female T2DM subjects. These results are in agreement with previous research, which has reported decreased serum SP-D levels 3 h after an acute session of physical exercise in healthy subjects as well as in those affected by rheumatoid arthritis disease [17]. Christensen et al. proposed a mechanism surrounding these findings that was attributed to cortisol levels fluctuations [17]. While the present investigation did not measure cortisol as a dependent variable, it is unlikely this hormone had a direct effect on the SP-D values that we reported, as cortisol varies minimally with the exercise intensity that was employed [25]. Additionally, in the current study blood samples were obtained 72 h after the last exercise session and this provided ample time for cortisol levels to return to baseline values [26].

In obese and/or T2DM subjects, previous studies have demonstrated systemic SP-D decreased levels [10, 12]. Indeed, evidence has demonstrated an association of low systemic SP-D levels with increased fat accumulation [10, 12] and decreased insulin sensitivity [10]. In our study, after 10 weeks, the insulin action and glycemic control of the C group deteriorated. On the other hand, subjects who performed chronic endurance training showed improvement in markers of glycemic control as evidenced by the reduced fasting glucose and HOMA-IR responses at the same time SP-D reduction. In this sense, Fernandez et al. have established that a normal insulin action is necessary for increasing systemic SP-D levels in response to an inflammation stimulate as in T2DM subjects systemic SP-D [10]. Also, according to results of an in vitro study, insulin can lead to a rise surfactant protein synthesis [27]. Thus, further investigation will be required to evaluate the positive functional consequences of exercise training-related changes in systemic SP-D, for instance systemic SP-D responses to an inflammatory stimulus.

A recent prospective human study suggests an association between SP-D and inflammation and atherosclerosis [4]. Low-grade systemic inflammation has been implicated in non-contagious diseases and is strongly associated with insulin resistance [28]. It is therefore possible that decreased serum SP-D levels following regular endurance training could result in decreasing low-grade chronic inflammation, leading to improvement of insulin resistance in individuals with T2DM.

Regarding obesity, it is generally known that proinflammatory cytokines such as IL-6, TNF-ɑ and innate immune mediator increase in the adipose tissue [29–31]. However, it seems that in addition to inflammation, SP-D plays a role in both energy metabolism and homeostasis; in this sense, results from previous studies have provided evidence that decreased systemic SP-D is associated with higher BMI in obese and T2DM subjects [10–12, 23]. Furthermore, Ortega et al. have shown that human adipose tissue expresses SP-D, however expression is decreased in an obese population [23]. Also, recent evidence revealed that SP-D knock-out mice were obese, presenting higher energy intake without increased energy expenditure [22]. In this sense, in human studies, Fernandez et al. have reported that weight loss led to decreased serum SP-D in obese women [10]. In our study, BMI and WHR decreased after the 10 week-period in the ET group, but these changes were not statically significant. In fact, these results show that chronic physical activity may lead to decreased systemic SP-D independently of changes in BMI in obese and/or T2DM. However, longer-term studies are needed to further evaluate the effects of exercise training on SP-D expression in adipose tissue of T2DM obese subjects.

With respect to contribution of SP-D in inflammatory responses in lung, it might influence metabolism by changing adipocytes production. Leptin is a hormone produced by adipose tissue which is implicated in insulin resistance, and may play a role in the etiology of T2DM. Leptin acts on the satiety center in the hypothalamus to suppress appetite, limit food intake and increase energy expenditure [32]. A leptin-resistant state has been demonstrated in obesity and obesity-related cardiovascular disease [33]. Also, there was a significant association between insulin resistance and serum leptin concentrations [34]. The exercise, particularly the aerobic type, has been shown to significantly lower leptin concentrations in T2DM and/or obese individuals [35, 36]. In the present study, leptin levels decreased significantly in ET if compared to control group, which could lead to glucose homeostasis improvement in the current study, once there are evidences indicating that leptin regulates the glucose homeostasis [37] and insulin sensitivity [38]. Therefore, it is possible that leptin decrease resulted in decreasing of circulating SPD in our study, being this phenomenon clinically relevant, because it allows us to suggest the use of this important hormone as a correlated biomarker of the pulmonary innate host defense of T2DM subjects.

Leptin represents body fatness and the balance between energy intake and expenditure [39]. Despite leptin concentration decreased in the ET group, BMI and WHR did not present significant changes. With regard to subjects of the present study (T2DM/free-living diet), it is possible suggest that the lower values of plasmatic leptin may be attributed to the higher amount of calorie expenditure during exercise training sessions. Also, some studies support a potential link between reduced leptin concentrations and elevated physical activity levels in patients with T2DM [36, 40]. We observed a significant increase in VO2max in the ET group after the intervention period and this is in agreement with a number of studies that have shown that regular exercise enhances VO2max and cardiovascular fitness in this population [41–44].

A limitation of this study was the nature of the participant population, i.e. the diabetic patients, which makes it difficult for researchers to control many independent factors other than obesity and diabetes itself, including genetics, which could possibly influence the findings. In this sense, Pueyo et al. reported that genetic variation in the coding region of SP-D are associated with increased insulin resistance and risk of T2DM development [24]. It is interesting to note that these associations are independent of systemic SP-D levels [24]. Also, these researchers suggested that stimulus factors such as weight gain, aging, and repeated usual-life infection, results in low-grade systemic inflammation which could reinforce SP-D gene polymorphisms that are associated with T2DM [24]. Thus, further studies are needed in order to better clarify a possible role of exercise in SP-D gene polymorphisms. Also, another limitation of our study which should not be ignored could be its small sample size. Therefore, further studies are needed to be conducted with bigger sample size.

## Conclusion

Endurance exercise training with improvement in aerobic fitness induced a significant reduction of serum leptin and SP-D levels in obese women with T2DM.

## Abbreviations

C: control
ET: endurance training
SP-A: surfactant protein A
SP-B: surfactant protein B
SP-C: surfactant protein C
SP-D: surfactant protein D
T2DM: type-2 diabetes *mellitus*
HRR: heart rate reserve
BMI: body mass index
WHR: waist to hip ratio
TC: total cholesterol
TG: triglyceride
HDL-c: high-density lipoproteins–cholesterol
HOMA-IR: Homeostatic Model Assessment-Insulin Resistance
RPE: rating of perceived exertion
ELISA: enzyme-linked immunosorbent assay
IL-6: interleukin 6
TNF-α: tumor necrosis factor alpha
